# Polyamine Catabolism in Plants: A Universal Process With Diverse Functions

**DOI:** 10.3389/fpls.2019.00561

**Published:** 2019-05-07

**Authors:** Wei Wang, Konstantinos Paschalidis, Jian-Can Feng, Jie Song, Ji-Hong Liu

**Affiliations:** ^1^ College of Horticulture, Henan Agricultural University, Zhengzhou, China; ^2^ Department of Agriculture, School of Agricultural Sciences, Hellenic Mediterranean University, Heraklion, Greece; ^3^ Key Laboratory of Horticultural Plant Biology, College of Horticulture and Forestry Sciences, Huazhong Agricultural University, Wuhan, China

**Keywords:** polyamine catabolism, polyamine oxidases, ROS, plant development, fruit ripening and senescence, abiotic and biotic stress

## Abstract

Polyamine (PA) catabolic processes are performed by copper-containing amine oxidases (CuAOs) and flavin-containing PA oxidases (PAOs). So far, several CuAOs and PAOs have been identified in many plant species. These enzymes exhibit different subcellular localization, substrate specificity, and functional diversity. Since PAs are involved in numerous physiological processes, considerable efforts have been made to explore the functions of plant CuAOs and PAOs during the recent decades. The stress signal transduction pathways usually lead to increase of the intracellular PA levels, which are apoplastically secreted and oxidized by CuAOs and PAOs, with parallel production of hydrogen peroxide (H_2_O_2_). Depending on the levels of the generated H_2_O_2_, high or low, respectively, either programmed cell death (PCD) occurs or H_2_O_2_ is efficiently scavenged by enzymatic/nonenzymatic antioxidant factors that help plants coping with abiotic stress, recruiting different defense mechanisms, as compared to biotic stress. Amine and PA oxidases act further as PA back-converters in peroxisomes, also generating H_2_O_2_, possibly by activating Ca^2+^ permeable channels. Here, the new research data are discussed on the interconnection of PA catabolism with the derived H_2_O_2_, together with their signaling roles in developmental processes, such as fruit ripening, senescence, and biotic/abiotic stress reactions, in an effort to elucidate the mechanisms involved in crop adaptation/survival to adverse environmental conditions and to pathogenic infections.

## Introduction

Polyamines (PAs) are small aliphatic amines present in all living cells. For more than 100 years in biology, they were misunderstood as “ptomaine or food poisoning” substances by toxicologists (Cohen et al., 1981). The largely known PAs in plants are putrescine (Put), spermidine (Spd), and spermine (Spm). In addition, cadaverine (Cad) and thermospermine (t-Spm), a Spm isomer, are also reported to exist in higher plants.

Polyamine homeostasis is determined by PA metabolism, conjugation, interconversion, chemical alteration and transport (Moschou and Roubelakis-Angelakis, 2014; Handa et al., 2018; Nguyen et al., 2018; Tiburcio and Alcazar, 2018; Podlesakova et al., 2019). Biochemical effects of PAs have been unraveled in many physiological processes, primarily in stability and function of proteins and nucleic acids (Handa et al., 2018), partly due to their positive charge that enables them to electrostatically interact with polyanionic molecules inside the cell. Polyamines correlate with numerous vital biochemical functions, including protein regulation (Takahashi and Kakehi, 2010; Sayas et al., 2019), regulation of chemiosmosis and photoprotection in chloroplasts (Ioannidis et al., 2016), ATP synthesis (Ioannidis et al., 2006), ion channeling (Pottosin et al., 2014a; Shabala et al., 2016), membrane fluidity (Paschalidis et al., 2010; Bleackley et al., 2014; Shabala et al., 2016; Dorighetto Cogo et al., 2018), and control of N/C balance (Moschou et al., 2012; Gupta et al., 2013; Majumdar et al., 2016). Exogenous PA application enhanced plant tolerance/resistance to several abiotic stress conditions, such as salinity, drought, water logging/flooding, osmotic stress, heavy metals, and extreme temperatures (Liu et al., 2006, 2015; Moschou et al., 2008c, 2012; Paschalidis et al., 2009a; Toumi et al., 2010; Moschou and Roubelakis-Angelakis, 2014; Gupta et al., 2016; Handa et al., 2018; Nguyen et al., 2018; Tiburcio and Alcazar, 2018; Pal et al., 2019; Yin et al., 2019). Polyamine application also enhanced tolerance to a few phytopathogenic infections *in planta*, such as *Alternaria alternata* (Estiarte et al., 2017), *Phytophthora capsici* (Koç, 2015), and *Pseudomonas viridiflava* (Rossi et al., 2015, 2018), and *in vitro*, such as *Fusarium* strains (Wojtasik et al., 2015) and *Sclerotinia sclerotiorum* (Garriz et al., 2003). The increase of host PA levels, either by using transgenic method or treatment with exogenous PAs, strongly decreased *in planta* growth of biotrophic pathogen *Pseudomonas viridiflava*, which was relieved by a PA oxidase (PAO) inhibitor (Marina et al., 2008). However, increase of leaf PA levels, by the same experimental approaches, led to increased necrosis *in planta* due to infection by *Sclerotinia sclerotiorum*, and the PA-induced increase of leaf necrosis after fungal infection was attenuated by inhibiting the activity of DAO and PAO (Marina et al., 2008). There is evidence that exogenous PA application modifies pathogenic responses depending on the strategy of the specific pathogen (Marina et al., 2008; Stes et al., 2011; Valdes-Santiago et al., 2012; Vilas et al., 2018).

Polyamines have crucial roles in a plethora of developmental procedures, including floral initiation and development (Liu et al., 2006, 2015; Liu and Moriguchi, 2007; Tavladoraki et al., 2016; Ahmed et al., 2017), leaf development and senescence (Kusano et al., 2008; Paschalidis et al., 2009b; Sobieszczuk-Nowicka et al., 2015; Sobieszczuk-Nowicka, 2017), fruit development and ripening (Liu et al., 2006; Liu and Moriguchi, 2007; Tsaniklidis et al., 2016; Fortes and Agudelo-Romero, 2018), and abiotic/biotic stress response (Alcazar et al., 2006, 2010; Moschou et al., 2008a; Liu et al., 2015, 2018; Montilla-Bascon et al., 2017).

Cellular PA levels are largely dependent on the dynamic regulation/balance among their biosynthesis, transport, and catabolism interchange. Polyamine biosynthesis has been thoroughly studied in the abovementioned physiological processes and a number of excellent literature reviews refer to their role (Kusano et al., 2008; Paschalidis et al., 2009a; Rangan et al., 2014; Liu et al., 2015; Majumdar et al., 2016; Fortes and Agudelo-Romero, 2018; Handa et al., 2018; Tiburcio and Alcazar, 2018; Wuddineh et al., 2018; Podlesakova et al., 2019). Nevertheless, there is a substantial lack of information on PA catabolism; so far, the enzymes involved in this process and the potential functions of their genes remain poorly characterized. As far as substrate specificity is concerned, it is well known that PAs are catalyzed by two major categories of amine oxidases, copper-containing amine oxidases (CuAOs) and flavin-containing PA oxidases (PAOs) (Cona et al., 2006), with cell type-specific functions in plant tissue/organ differentiation and development (Tavladoraki et al., 2016).

Emerging evidence suggests that PA catabolism plays a critical signaling role in a variety of cellular and developmental processes in all organisms, mediated *via* regulation of their homeostasis in reaction to intercellular and/or intracellular signs, as developmentally generated by abiotic and/or biotic alarms. In an effort to elucidate the underlined biological mechanisms, the latest advances are updated here on the function of CuAOs and PAOs, as sources of bio-reactive products, such as H_2_O_2_, during developmental processes with emphasis in fruit ripening and senescence, and, moreover, in abiotic/biotic stress reactions. The present approach might help in unraveling the role/use of the PA catabolic pathway in plants as a focus area for innovative stress resistance/tolerance approaches.

## Advance in Polyamine Catabolism Research

### Copper-Containing Amine Oxidases in Polyamine Catabolism

Generally, in terms of substrate specificity, CuAOs exhibit strong preference for diamines (Put and Cad), and mainly catalyze their oxidation at primary amino groups, thus generating 4-aminobutanal, H_2_O_2_, and ammonia (Alcazar et al., 2010; Moschou et al., 2012). However, it has been demonstrated that some CuAOs in *Arabidopsis* also catalyze the oxidation of Spd (Planas-Portell et al., 2013). Recently, CuAO genes from apple (*Malus domestica*) exhibited different substrate preferences, with MdAO1 displaying elevated catalytic efficiency for 1,3-diaminopropane, Put, and Cad, whereas MdAO2 consumed only aliphatic and aromatic monoamines, comprising 2-phenylethylamine and tyramine (Zarei et al., 2015). Plant CuAOs usually exist at increased levels in dicot plants (Cona et al., 2006). Their genes have been identified in several species, as, for example, *Arabidopsis* (Møller and McPherson, 1998; Planas-Portell et al., 2013), chickpea (Rea et al., 1998), pea (Tipping and McPherson, 1995), tobacco (Paschalidis and Roubelakis-Angelakis, 2005b; Naconsie et al., 2014), apple (Zarei et al., 2015), grapevine (Paschalidis et al., 2009b), and sweet orange (Wang et al., 2017). *Arabidopsis* has at least ten recognized *CuAO* genes, however, only five of them (*AtAO1*, *AtCuAO1*, *AtCuAO2*, *AtCuAO3*, and *AtCuAO8*) have been characterized at protein level (Møller and McPherson, 1998; Planas-Portell et al., 2013; Ghuge et al., 2015; Groβ et al., 2017). The apple genome contains five putative *CuAO* genes with two of them (*MdAO1* and *MdAO2*) being identified at protein level (Zarei et al., 2015) and, recently, eight putative *CuAO* genes were reported in sweet orange (Wang et al., 2017).

As far as subcellular localization is concerned, plant CuAOs are separated into two groups (Zarei et al., 2015). The first group includes CuAOs that are typical extracellular proteins which contain an N-terminal signal peptide. Until now, seven CuAO members of the first group have been reported comprising *Pisum sativum* (PsCuAO), apple (MdAO2), *Arabidopsis* (AtAO1 and AtCuAO1), and sweet orange (CsCuAO4, CsCuAO5, and CsCuAO6) (Tipping and McPherson, 1995; Møller and McPherson, 1998; Planas-Portell et al., 2013; Zarei et al., 2015; Wang et al., 2017). The second group includes CuAOs localized in peroxisomes, containing a C-terminal peroxisomal targeting signal 1 (PTS1). At present, seven CuAO members of the second group have been reported, including two CuAOs from *Arabidopsis* (AtCuAO2 and AtCuAO3), two from tobacco (NtMPO1 and NtCuAO1), one from apple CuAO (MdAO1), and two from sweet orange (CsCuAO2 and CsCuAO3) (Planas-Portell et al., 2013; Naconsie et al., 2014; Zarei et al., 2015; Wang et al., 2017).

### Polyamine Oxidases as Terminal and Back-Conversion Reaction Types in Polyamine Catabolism

In contrast to CuAO, in terms of substrate specificity, PAOs exhibit strong affinity for Spd, and Spm, as well as their derivatives (Alcazar et al., 2010). According to their functions in PA catabolism and subcellular localization, plant PAOs can be classified into two classes. The first class of PAOs (PA terminal catabolism reaction type) performs the oxidation and decomposition of Spd and Spm producing H_2_O_2_, 1,3-diaminopropane (DAP), and 4-aminobutanal (Spd catabolism) or N-(3-aminopropyl)-4-aminobutanal (Spm catabolism) (Cona et al., 2006; Angelini et al., 2010; Moschou et al., 2012; Tavladoraki et al., 2016; Bordenave et al., 2019). On the other hand, the second group (PA back-conversion reaction type) includes PAOs that catalyze the PA back-conversion reactions which convert Spm to Spd and Spd to Put (Moschou et al., 2012; Tavladoraki et al., 2016; Takahashi et al., 2018), in a reverse reaction of PA synthesis and produces 3-aminopropanal and H_2_O_2_. Although PAOs occur at high levels in monocot plants (Sebela et al., 2001), until now, *PAO* genes have been characterized in both monocots and dicots, including maize (Tavladoraki et al., 1998; Cervelli et al., 2000, 2006), rice (Ono et al., 2012), barley (Smith and Davies, 1985; Cervelli et al., 2006), *Arabidopsis* (Fincato et al., 2011), tobacco (Paschalidis and Roubelakis-Angelakis, 2005b; Yoda et al., 2006), grapevine (Paschalidis et al., 2009b), poplar (Tuskan et al., 2006), apple (Kitashiba et al., 2006), sweet orange (Wang and Liu, 2015, 2016), *Brachypodium* (Takahashi et al., 2018), tomato (Ono et al., 2012; Chen et al., 2016; Sagor et al., 2017; Hao et al., 2018), and upland cotton (Chen et al., 2015). So far, only six *PAO* genes that belong to the first group have been identified. The best characterized *PAO* gene of the first group is the maize *PAO* gene (*ZmPAO*) (Tavladoraki et al., 1998; Cona et al., 2006) and *PAO* genes from barley (*HvPAO1* and *HvPAO2*), rice (*OsPAO7*), sweet orange (*CsPAO4*), and *Brachypodium* (*BdPAO2*), which are proved to catalyze the PA terminal catabolism (Smith and Davies, 1985; Liu et al., 2014a; Wang et al., 2016; Takahashi et al., 2018). In contrast, most of the identified plant *PAO* genes belong to the second group. All of the five existing *PAO* genes in *Arabidopsis* (*AtPAO1*–*AtPAO5*) catalyze the PA back-conversion reactions (Tavladoraki et al., 2006; Kamada-Nobusada et al., 2008; Moschou et al., 2008c; Fincato et al., 2011; Ahou et al., 2014). In the rice genome, four (*OsPAO1*, *OsPAO3*, *OsPAO4*, and *OsPAO5*) out of seven (*OsPAO1–OsPAO7*) existing *PAO* genes execute the PA back-conversion reactions (Ono et al., 2012; Andronis et al., 2014; Liu et al., 2014b; Zarza et al., 2017). Similarly, in the tomato genome, four (SlPAO2, SlPAO3, SlPAO4, and SlPAO5) out of seven (SlPAO1–SlPAO7) existing PAO genes are suggested to execute the PA back-conversion reactions (Hao et al., 2018). On the other hand, six putative *PAO* genes have been identified in sweet orange and only one of them (*CsPAO3*) is demonstrated to catalyze the PA back-conversion reactions (Wang et al., 2016) and, of the 12 putative PAO genes (*GhPAO1–GhPAO12*) recognized in upland cotton, only one (*GhPAO3*) is verified to be implicated in the back-conversion pathway (Chen et al., 2017). To date, in terms of subcellular localization, all of the reports support that the PA terminal catabolic pathway is specifically activated in the apoplastic compartments (extracellularly), whereas the PA back-conversion pathway mainly occurs in the intracellular space (peroxisomes).

Beyond their functional/subcellular localization, in terms of either the terminal or the back-conversion type, PAOs exhibit further individual substrate specificities. The AtPAO1 only catalyzed the oxidation of Spm, but not Spd (Tavladoraki et al., 2006), while AtPAO3 preferred Spd as substrate instead of Spm (Moschou et al., 2008c). However, the AtPAO2 and the AtPAO4 present similar preference for both Spd and Spm (Fincato et al., 2011). Differently, AtPAO5 only uses t-Spm as its substrate and catalyzes the back-conversion of t-Spm to Spd (Kim et al., 2014). Furthermore, PAOs also exhibit individual reaction conditions, as, for example, they present different optimal pH values and temperature upon catalyzing different substrates. The optimal pH of catalytic activity for AtPAO2 is 7.5 towards both Spd and Spm, while the optimal pH for AtPAO4 catalytic activity towards Spd and Spm is 8.0 and 7.0, respectively (Fincato et al., 2011). In adddition, for CsPAO4 catalytic activity the optimal pH was 7.0 towards Spd and 8.0 towards Spm (Wang and Liu, 2016).

## Polyamine Catabolism in Plant Development

Increasing studies report that PA catabolism is directly involved in plant development. Several evidence suggests that PA oxidation in the apoplast together with the generated reactive oxygen species (ROS) are involved in programmed cell death (PCD) and xylem differentiation (Corpas et al., 2019; Podlesakova et al., 2019). As early as 1998, Møller and McPherson found that *AtCuAO* localization in root xylem tissues is preceding and overlays with the synthesis of lignin in *Arabidopsis* (Møller and McPherson, 1998), and the PAO-generated apoplastic H_2_O_2_ levels considerably contribute to *Zea mays* leaf blade elongation (Rodriguez et al., 2009). In addition, the perturbation of PA catabolism by overexpressing the *ZmPAO* gene, as well as by down-regulating the S-adenosyl methionine decarboxylase (*SAMDC*) gene *via* RNA interference, in tobacco, promotes vascular cell differentiation and induces PCD in root cap cells (Moschou et al., 2008b; Tisi et al., 2011). Recently, the *AtPAO5* has been reported to participate in the tightly controlled interplay between auxins and cytokinins, which are necessary for proper xylem differentiation (Alabdallah et al., 2017), and to regulate *Arabidopsis* growth through t-Spm oxidase activity (Kim et al., 2014).

Other studies suggest that PAs, along with ROS derived by their oxidation, control ion channeling in plant cells throughout normal and stress conditions, by affecting the plasma membrane ion transporting or acting as second messenger molecules (Pegg, 2014; Pottosin et al., 2014b). It has been reported that the Spd oxidase-produced H_2_O_2_ controls pollen plasma membrane hyperpolarization-activated Ca(^2+^)-penetrable canals and pollen tube growth (Wu et al., 2010). In *Arabidopsis thaliana*, differences in expression patterns are revealed for all of the AtPAO gene family members, as *AtPAO1* was mainly found in the transition area among meristems and elongation root regions, as well as in anther tapetum, and *AtPAO2* was most expressed in the pollen, quiescent center and columella initials, whereas AtPAO3 was predominantly identified in pollen, columella and guard cells. In addition, *AtPAO5* was specifically expressed in the root vascular system and in hypocotyls (Fincato et al., 2012). Moreover, the gene structure of *AtPAO5* was quite different from the other four *AtPAO* genes (Fincato et al., 2011). Its expression was detected during various growth stages, with the highest expression being observed in flowers, especially in sepals (Takahashi et al., 2010). AtPAO5 is classified as a cytosolic Spm oxidase/dehydrogenase protein undergoing proteasomal control (Ahou et al., 2014), that controls *Arabidopsis* growth *via* t-Spm oxidase activity (Kim et al., 2014; Liu et al., 2014d), while the rice OsPAO1 is a functional ortholog of AtPAO5 (Liu et al., 2014d) and the rice OsPAO7 is involved in lignin synthesis in anther cell walls (Liu et al., 2014c).

## Polyamine Catabolism in Fruit Ripening and Senescence

Fruits usually keep higher PA levels at early developmental stages and are followed by a continuing decrease thereafter, especially at ripening stage (Fortes and Agudelo-Romero, 2018). This phenomenon has been reported in both climacteric and non-climacteric fruits, such as apple (Biasi et al., 1988), avocado (Kushad et al., 1988), peach (Liu and Moriguchi, 2007; Ziosi et al., 2009), mango (Malik and Singh, 2004), olive (Gomez-Jimenez et al., 2010), tobacco (Paschalidis and Roubelakis-Angelakis, 2005a), strawberry (Guo et al., 2018), raspberry (Simpson et al., 2017), oil palm (Teh et al., 2014), tomato (Rastogi and Davies, 1991; Tassoni et al., 2006; Mattoo et al., 2007; Van de Poel et al., 2013; Tsaniklidis et al., 2016; Liu et al., 2018), and grapevine (Paschalidis et al., 2009b; Agudelo-Romero et al., 2013; Fortes et al., 2015). As PA contents largely depend on the balance between anabolism and catabolism, it is necessary to unravel this balance during fruit ripening.

Although the precise roles of PA catabolism in fruit ripening are poorly understood, current studies reveal their tight interplay. High expression levels of CuAOs and PAOs during fruit ripening denote the involvement of PAs in associated physiological processes. For example, the free and conjugated PAs dramatically decrease during grape ripening, together with an up-regulation of the CuAO and PAO genes/enzymes and an increase of the H_2_O_2_ content (Agudelo-Romero et al., 2013), as well as an increase in γ-aminobutyric acid (GABA), a major product of PA catabolism (Fortes et al., 2015). These data suggest that increased PA oxidation might lead to decrease in PA titers. As PAO-derived ROS usually act as secondary messengers, the up-regulation of CuAOs/PAOs during ripening might establish an adequate ROS source for signaling actions driving to ripening hastening. In peach fruit, a jasmonate-induced ripening delay was closely related to increased PA levels (Ziosi et al., 2009). A few studies have unraveled strong indications for the interactions among PAs, PAO-derived products, and hormones, such as abscisic acid (ABA), cytokinins, auxins, and ethylene, aiming on their coordinated action in signaling pathways of several physiological processes, like fruit ripening and stress response (Podlesakova et al., 2019) (Agudelo-Romero et al., 2013). Inhibition of PA catabolism in grape with guazatine, a potent inhibitor of PAO activity, led to profound changes in amino acids, carbohydrates, and hormonal metabolism (Agudelo-Romero et al., 2013).

## Polyamine Catabolism in Abiotic Stress

Increasing evidences have showed that the plant PA catabolism is involved in various abiotic stresses responses, especially in salinity. Previously, it has been reported that GABA generated by CuAO-mediated PA oxidation exerts a substantial role in salinity stress response (Su et al., 2007). The PAOs exerting multifaceted roles on plant growth and salt stress response have been identified in, among others, tobacco, grapevine, sweet orange, tomato, and *Arabidopsis* (Moschou et al., 2008b; Paschalidis et al., 2010; Fincato et al., 2011; Wang and Liu, 2016; Gemes et al., 2017; Hao et al., 2018). Salinity induces tobacco cells to secrete exodus of Spd to the apoplast, where it is oxidized by PAO, thus generating abundant H_2_O_2_ and leading to enhanced PCD (Moschou et al., 2008a,b). A *PAO* gene of sweet orange (*CsPAO4*) has been further characterized functioning in PA terminal catabolism and playing an important role against salinity (Wang and Liu, 2016). This *CsPAO4* was overexpressed in tobacco, which significantly promoted the germination of transgenic seeds, while prominently inhibited the vegetative growth and root elongation of transgenic plants under salinity (Wang and Liu, 2016). The PAO activity provided a significant apoplastic production of ROS, which partly contributed to the maize leaf blade elongation under salt stress (Rodriguez et al., 2009). On the other hand, the peroxisomal AtPAO5 loss-of-function mutation in *Arabidopsis thaliana* exhibits constitutively higher t-Spm levels and activates metabolic and transcriptional reprogramming promoting salinity stress protection (Zarza et al., 2017). Moreover, PAO inhibitor treatment significantly decreased the H_2_O_2_ and NO production in tomato under salinity (Takacs et al., 2016), which indicates that PAO may contribute to H_2_O_2_ and NO production in order to cope with salinity and that the terminal activities of CuAO and PAO might play a role in cell death induction under lethal salt stress. The PA catabolism is also involved in many other abiotic stress responses, among others, in improved thermotolerance in *Nicotiana tabacum* by underexpressing the apoplastic PA oxidase (Mellidou et al., 2017), in aluminum-induced oxidative stress of wheat (Yu et al., 2018), in selenium-induced H_2_O_2_ production in *Brassica rapa* (Wang et al., 2019), and in wound-healing by producing the necessary H_2_O_2_ for suberin polyphenolic domain and lignin synthesis catalyzed by peroxidase (Angelini et al., 2008).

## Polyamine Catabolism in Pathogen Response

Plants have developed a series of strategies to thwart pathogen attack (Vilas et al., 2018). The production of ROS is one of the defense responses against pathogen attack. Hydrogen peroxide may act either as an antimicrobial means preventing pathogen from growing or contributing as a signaling molecule, which induces the activation of protecting genes (Corpas et al., 2019).

As the terminal catabolism of PAs is followed by the generation of H_2_O_2_, PA catabolism is, thus, involved in pathogen defense response. The ornithine decarboxylase (ODC) activity increased 20-fold during the hypersensitive response (HR) to tobacco mosaic virus (TMV) infection; however, the levels of Put, Spd, and Spm were not greatly altered, as expected (Negrel et al., 1984), while the activities of arginine decarboxylase (ADC), ornithine decarboxylase (ODC), and CuAO were all obviously increased (Marini et al., 2001). In addition, the PAO expression level and PA titers were also increased in tobacco plants resistant to TMV (Yoda et al., 2003, 2006), suggesting that both PA biosynthesis and catabolism are activated in the host during pathogen infection, where appropriate.

The H_2_O_2,_ resulted from increased activities of CuAO and PAO, might be the cause for the HR observed in barley after powdery mildew infection (Cowley and Walters, 2002). The increase of host PA levels limited bacterial growth, while inhibition of the PAO host enzymes increased the infection (Marina et al., 2008). It has been reported that DAO and PAO activities might play role in promoted defense against biotrophic or hemibiotrophic pathogens. However, these activities enhanced the infection of necrotrophic pathogens (Marina et al., 2008; Yoda et al., 2009; Moschou et al., 2009a). Similarly, the accumulation and further oxidation of free PAs was detected in the apoplast of tobacco leaves during tobacco defense against infection by microorganisms with contrasting pathogenesis strategies (Marina et al., 2008). This response affected the pathogen’s ability to colonize host tissues and was detrimental for plant defense against necrotrophic pathogens, but it might be beneficial for plant defense against biotrophic pathogens because the former fed on necrotic tissue while the latter depended on living tissue for successful host colonization (Marina et al., 2008). Therefore, apoplastic PAs were suggested to play significant roles in plant-pathogen interactions and lead to significant changes in host susceptibility to different kinds of pathogens through regulation of host PA levels, particularly in the leaf apoplast (Marina et al., 2008). Similarly, tobacco plants overexpressing a *ZmPAO* unraveled a preinduced disease tolerance against the biotrophic bacterium *Pseudomonas syringae* pv *tabaci* and the hemibiotrophic oomycete *Phytophthora parasitica var nicotianae* (Moschou et al., 2009a), showing a critical role for a PAO-generated H_2_O_2_ apoplastic barrier for these fungi and bacteria. The PA catabolism also contributed to a resistance state through modulation of the immune response in grapevine following osmotic stress and/or after *Botrytis cinerea* infection (Hatmi et al., 2018). The pretreatment of stressed berries with appropriate inhibitors of DAO and PAO further increased PA level and greatly lowered defense responses, leading to higher susceptibility to *B. cinerea* (Hatmi et al., 2018). It is evident that the host PA apoplastic catabolism and the mediated H_2_O_2_ accumulation play an important signaling role in plant-pathogen interactions. However, the specific mechanisms of PA catabolism against plant resistance to pathogens are often more complicated. Further research is needed to clarify the exact role of PA catabolism in biotic stress resistance, in an effort to help plants cope with adverse environmental conditions and survive.

## Polyamine Catabolism and H_2_O_2_ in Abiotic and Biotic Stress Responses

Stress conditions are accompanied by ROS accumulation and induce a composite signaling system recognized by endogenous plant cell sensors and transferred *via* secondary messengers to kinases, which lead to differentiations in gene expressions and related metabolites by means of the corresponding transcription factors in a plethora of processes identified as stress responses (Skopelitis et al., 2006; Waszczak et al., 2018).

In addition to several pathways, as, for example, photorespiration and electron transferring in chloroplasts and mitochondria, ROS are produced by apoplastic enzymes or enzymes that have different subcellular localization (Moschou and Roubelakis-Angelakis, 2014; Waszczak et al., 2018; Bordenave et al., 2019). The NADPH oxidase (Papadakis et al., 2005; Papadakis and Roubelakis-Angelakis, 2005; Andronis et al., 2014; Gemes et al., 2016), peroxidases (Papadakis et al., 2005; Paschalidis and Roubelakis-Angelakis, 2005b), oxalate oxidase (Angelini et al., 2008), xanthine dehydrogenase (Zarepour et al., 2010), and PAOs (Paschalidis and Roubelakis-Angelakis, 2005b; Moschou et al., 2008a,b,c; Paschalidis et al., 2009a, 2010; Takahashi et al., 2010; Gupta et al., 2016; Tavladoraki et al., 2016; Hao et al., 2018; Wu et al., 2018; Corpas et al., 2019) are included in these pathways, depending on each specific occasion.

Polyamines, as key compounds in plant physiology, are involved in this stress-signaling scheme, playing essential roles in the control of plant stress tolerance (Moschou and Roubelakis-Angelakis, 2014). Furthermore, numerous protein kinases are transcriptionally or posttranscriptionally regulated by PAs (Moschou and Roubelakis-Angelakis, 2014). Almost 3.5 centuries since their discovery – 1,678 in human semen – PAs still remain fundamental research interests, as they are widely implicated in a plethora of developmental and stress signaling responses. Proteomic and transcriptomic analyses on the PA-stress interplay and identification of over- or underexpressed key related genes, among others, ADC, ODC, SAMDC, Spd synthase (SPDS), Spm synthase (SPMS), CuAOs, and PAOs (Liu et al., 2006, 2015; Liu and Moriguchi, 2007; Tanou et al., 2014; Corpas et al., 2019) may offer a new insight into the molecular mechanisms controlling stress responses. Polyamines partially reversed the NaCl-induced phenotypic and physiological disturbances and systematically up-regulated the expression of PA biosynthesis (ADC, SAMDC, SPDS, and SPMS) and catabolism (DAO and PAO) genes, reprograming the oxidative and nitrosative status and the proteome of citrus plants exposed to salinity stress (Tanou et al., 2014). Recent transcriptomic analyses of the effect of Spd or norspermidine on *Arabidopsis* indicate up-regulation of the response to heat stress and denatured proteins, inhibiting protein ubiquitylation, both *in vivo* and *in vitro*, and this interferes with protein degradation by the proteasome, a situation known to deplete cells of amino acids (Sayas et al., 2019). Furthermore, by *in situ* RNA–RNA hybridization approaches, the spatial contribution of ODC1, 2; ADC2; and CuAO gene transcripts has been largely elucidated in developing tomato fruits in order to decode the potential connection of PA anabolism/catabolism to developmental processes, like fruit ripening (Tsaniklidis et al., 2016).

Polyamines may further alleviate the unfavorable stress effects by activating the antioxidant machinery (Podlesakova et al., 2019). Spd and Spm, and to a lesser extent, Put, inhibit NADPH-oxidase, whereas Put prevents the induction of PCD (Papadakis and Roubelakis-Angelakis, 2005; Andronis and Roubelakis-Angelakis, 2010; Andronis et al., 2014). Abiotic and biotic stress may cause radical alterations in PA metabolism. Several model systems, like *Arabidopsis thaliana*, have helped in deciphering the role of PAs and elucidating their metabolic paths (Gupta et al., 2016). The preservation of an appropriate balance of the PA catabolic pathways with the H_2_O_2_ dual role under normal and stress conditions has helped in illuminating the plant adaptation mechanisms (Paschalidis and Roubelakis-Angelakis, 2005b; Paschalidis et al., 2010; Gupta et al., 2016). ABA is an upstream signal for the induction of the polyamine catabolic pathway in the apoplast of grapevine, thus, amine oxidases are producing H_2_O_2_ which signals stomatal closure (Paschalidis et al., 2010). When the titers of H_2_O_2_ are below a threshold, expression of tolerance effector genes is induced, while when it exceeds this threshold, the PCD syndrome is induced (Paschalidis et al., 2010). Polyamines also increase nitric oxide and ROS in guard cells of *Arabidopsis thaliana* during stomatal closure (Agurla et al., 2018) and during growth inhibition in *Triticum aestivum* L seedlings (Recalde et al., 2018). In addition, the redox gradient across plasma membranes may play an essential role in climate changes, as a redox signaling regulator (Gupta et al., 2016).

Plant life and stress go hand-to-hand. During growth, in order to overcome abiotic stress conditions, plants develop a remarkable organ/tissue/age-specific PA-related phenotypic plasticity (Paschalidis and Roubelakis-Angelakis, 2005a). Under favorable conditions, a balanced hypogeous and hypergeous PA homeostasis is critical to allow constant water/nutrient uptake and photosynthetic flux, respectively. For example, PA genes/metabolites may contribute to an accurate adaptation of the shift between advancement in cell cycle/cell division, that pushes the growth of very young root/shoot primordia toward cell expansion, differentiation, and lignification (Paschalidis and Roubelakis-Angelakis, 2005b; Paschalidis et al., 2009a). On the contrary, plant growth under abiotic stress might, among other effects, wound/wilt the leaf surface or increase evaporation, rendering plant susceptibility. In this case, plants constantly examine whether or not the environmental signals are favorable for their development/growth, and might redirect a PA-associated phenotypic plasticity, involving Η_2_Ο_2_, the product of PA catabolism, either for growth or for stress adaptation, e.g., *via* spermidine-mediated stomatal closure (Paschalidis et al., 2009b, 2010). It is also specified that the seriousness/type of reaction (s) to (a)biotic stress is a cell/tissue/organ/age-specific route, related to PA catabolism (Paschalidis et al., 2009b, 2010). The assessment of the antioxidant genes/machinery, along with the photosynthetic factors, the intracellular cation titers, and the PA interplay in over/underexpressing ZmPAO plants under prolonged/varying salinity (Gemes et al., 2016, 2017) and heat (Mellidou et al., 2017) stress have highlighted a plant ontogenetic stage-specific role for PA oxidase and Η_2_Ο_2_ during plant developmental reactions to (a)biotic stress conditions.

**Figure 1:**
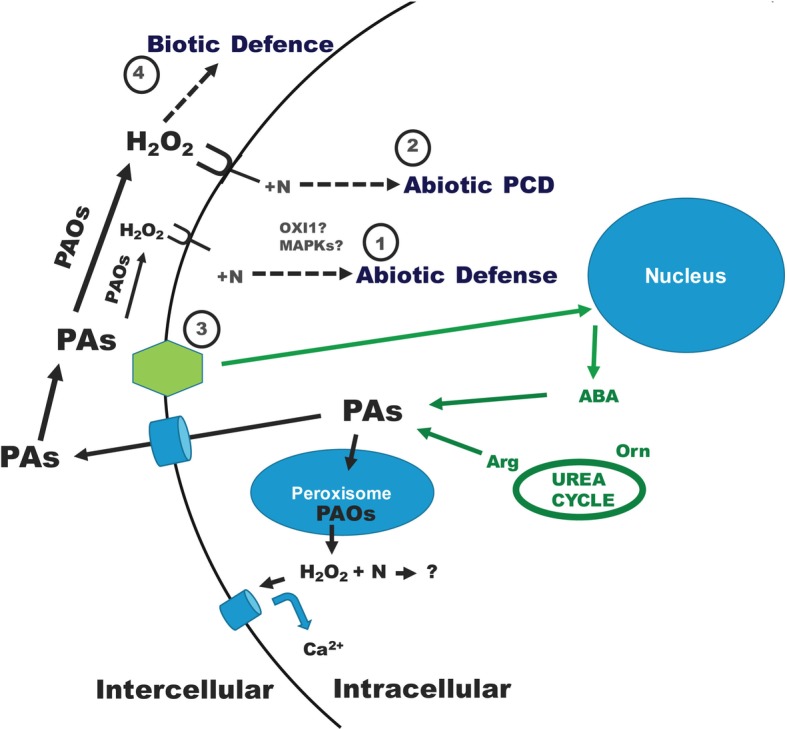
Dual polyamine catabolic model for signaling plant abiotic and biotic stress defense. A stress signal is recognized by numerous sensors and is transferred by several cellular biochemical pathways. Abiotic and biotic stresses result in ROS production. The stress-signaling pathway gives also rise to intracellular PAs, which are secreted/oxidized in the apoplast by PAOs in order to supply H_2_O_2_ and several N compounds. Hydrogen peroxide and N molecules may involve further reactions, including, among others, mitogen-activated protein kinases (MAPKs) and Oxidative Signal Inducible 1 (OXI1) pathways (Rentel et al., 2004; Moschou et al., 2009a,b, 2012; Toumi et al., 2010; Moschou and Roubelakis-Angelakis, 2014; Gupta et al., 2016; Podlesakova et al., 2019). Under abiotic conditions, according to the H_2_O_2_ level created: ① when low (H_2_O_2_ below a specific threshold), it is powerfully scavenged leading to abiotic defense or ② when high (H_2_O_2_ over a specific threshold), it cannot be efficiently scavenged and PCD is caused. The abiotic tolerance stress signal (③) is received by plants generating essential signal molecules like ABA that are involved in an augmentation of PA synthesis rendering tolerance in plants (Toumi et al., 2010). Under biotic stress conditions, mostly Spm is secreted in the apoplast and oxidized by the respective enriched PAO, causing a H_2_O_2_ buildup (④, biotic defense) that protects plants from phytopathogenic bacteria. In the scavenging process, antioxidant enzymes are involved, such as ascorbate peroxidase (APX), in a procedure rendering defense reactions. The implication of PA oxidation to H_2_O_2_ production is not only a matter of apoplastic or cytoplastic PAOs. Polyamines are also back-converted in peroxisome, with the parallel generation of H_2_O_2_ and nitrogenous substances. Peroxisomally produced H_2_O_2_ might trigger Ca^2+^-penetrable canals (Wu et al., 2010; Moschou et al., 2012; Zepeda-Jazo and Pottosin, 2018; Corpas et al., 2019). However, the N compounds generated as a result of the PA back-conversion path are not yet elucidated. Further details are found in the text.

During abiotic stress conditions, PAs (mainly Spd) are secreted in the apoplast and oxidized by PAOs (they refer to both CuAOs and PAOs, but, for simplicity, they are depicted only as PAOs, throughout the model presentation) (Figure 1), resulting in PA catabolism intermediates. The level of PAO-mediated Spd oxidation results in: (①) moderate apoplastic PAO oxidizing Spd at a small percentage producing modest (beneficial) H_2_O_2_ (and 1,3-diaminopropane) contents, that act as signaling molecules, inducing a ROS-dependent protective pathway, thus triggering abiotic stress tolerance reactions; (②) high apoplastic PAO, over a specific threshold, oxidizing Spd considerably faster, producing high (harmful) H_2_O_2_ levels, and resulting in down-regulation of pro-survival genes and execution of a specific PCD pathway in plants under abiotic stress conditions (Figure 1; Moschou et al., 2008b; Moschou and Roubelakis-Angelakis, 2014; Gupta et al., 2016; Corpas et al., 2019).

A possible scenario below may be postulated in order to explain the stress signaling/defense. Abiotic stress induces the production of intracellular and extracellular H_2_O_2_, higher PAs, and second messengers like Ca^2+^ (Wu et al., 2010). The higher PA levels, when oxidized, generate additional H_2_O_2_ that activates the plant antioxidant machinery. Indeed, under salt stress conditions, with increased levels of endogenous PAs induced by exogenously applied Spd, PAO activity is further enhanced, thus contributing to H_2_O_2_ accumulation, subsequently inducing enhanced antioxidant defense, which is helpful for growth (Wu et al., 2018). A cold-responsive ethylene-responsive factor from *Medicago falcata* was demonstrated to confer cold tolerance by upregulating polyamine turnover, antioxidant protection, and proline accumulation (Zhuo et al., 2018). ABA endogenous contents are also activated by stress, which may trigger ROS-related routes involving PAs (Gupta et al., 2016). A well-organized protection mechanism comprising of PAs, Ca^2+^, ABA, and H_2_O_2_ coordinates an adaptation response of plants to stress (Figure 1; Skopelitis et al., 2006; Moschou et al., 2008b; Paschalidis et al., 2010; Toumi et al., 2010; Moschou and Roubelakis-Angelakis, 2014; Gupta et al., 2016; Majumdar et al., 2016; Gemes et al., 2017; Handa et al., 2018).

Under abiotic stress conditions, ABA triggers the PA machinery in tolerant/sensitive grapevine genotypes (Toumi et al., 2010). The abiotic tolerance stress signal (③) is received by plants generating essential signal molecules like ABA that are involved in an augmentation of PA synthesis (Figure 1). Tolerant plants showed higher PA synthesis, as compared with the sensitive, giving rise to higher PA levels (Toumi et al., 2010). Regardless the genotype competence in withstanding stress, PAs follow the secretion way and are oxidized in the apoplast by PAOs (Paschalidis et al., 2010; Toumi et al., 2010). In this way, higher intracellular PA titers and higher PA synthesis, together with the apoplastic PAO-derived H_2_O_2_, are participating in a “positive feedback loop” helping to maintain homeostasis and enhance tolerance through activation of further defense mechanisms. On the contrary, lower PA titers/anabolism enhance PCD syndrome (Paschalidis et al., 2010; Toumi et al., 2010; Gemes et al., 2017).

This model/hypothesis elucidates the role of mostly the intercellular PAs. In abiotic-induced PCD of down-regulated SAMDC tobacco plants, the cellular Spd and Spm levels were reduced, but, unexpectedly, these plants showed similar, to the wild type, PA levels and oxidation in the apoplast (Moschou et al., 2008b). The plants with silenced SAMDC unravel a PA-dependent trade-off between growth and tolerance reactions (Mellidou et al., 2016) and the stimulation of the ADC pathway acts as a positive feedback loop to maintain the PA homeostasis (Toumi et al., 2010).

A biotic stress-induced increase in PAO gene/enzyme occurred in overexpressing PAO tobacco plants infected by *Pseudomonas syringae* pv *tabaci* (Moschou et al., 2009a). Under biotic stress conditions, mostly Spm is secreted in the apoplast and oxidized by the respective enriched PAO, causing a H_2_O_2_ buildup (④, biotic defense) that protects plants from phytopathogenic bacteria (Figure 1). In this context, overexpressing PAO plants reveal a preinduced tolerance against diseases, such as the biotrophic bacterium *Pseudomonas syringae* pv *tabaci* and the hemibiotrophic oomycete *Phytophthora parasitica* var *nicotianae* (Moschou et al., 2009a). PAO and DAO activities promote defense against biotrophic or hemibiotrophic pathogens and, by contrast, these activities favor the spread of the lesions provoked by necrotrophic pathogens (Marina et al., 2008; Yoda et al., 2009; Moschou et al., 2009a). Oxidation of others polyamines, such as t-Spm, is also involved in response to pathogenic bacteria, increasing *Arabidopsis* resistance to *Pseudomonas viridiflava* (Marina et al., 2013). This is probably related to the ability of plant PAOs to oxidize t-Spm in a wide range of tissues and organs, as occurs when other PAs such as Spm are accumulated throughout the plant (Marina et al., 2013). In addition to that, pathogens activate their own and the plant PA metabolism during the compatible interaction between tomato and *Pseudomonas syringae* (Vilas et al., 2018). This activation results in the accumulation of Put in whole leaf tissues, as well as in the apoplastic fluids, which is explained by the induction of its synthesis in plant cells and also on the basis of its excretion by bacteria (Vilas et al., 2018). *Ralstonia solanacearum* also produces abundant Put, acting as a virulence metabolite and accelerating wilt disease, possibly reducing ROS in the host (Lowe-Power et al., 2018). The present dual abiotic and biotic stress protection scheme may represent an innovative route for generating tolerant transgenic plants to a variety of environmental and phytopathogenic stress factors.

## Polyamines Act as Orthodox-Concerters or Stress-Relievers

During development, several molecules exist inside common plant tissues in normal environmental and phytopathogenic-free states, concerting an orthodox plant behavior. However, as soon as normal conditions are substituted by stressful ones, these molecules begin to work as stress-relievers. In this work, PAs are suggested to work as such molecules, i.e., as “orthodox-concerters” under normal conditions and as “stress-relievers” under stressful ones. Polyamines have established duties inside plants; however, when they are found in adverse conditions, they may reveal novel functions, not expected until that time. Polyamines, PA oxidases, and the generated H_2_O_2_ all have specific roles in sustaining plant developmental procedures, such as fruit ripening and senescence. Furthermore, in this work, the role for the concerted action of PA catabolism and its products, in reaction to both abiotic and biotic stress are discussed. The PA oxidation will surely remain a fascinating area for scientific examination, as its concerted action with the generated H_2_O_2_ is shown to classify specific stressful parameters and build an effective defense device.

## Conclusion

To date, many attempts have been made to investigate the roles of PA catabolism in plant growth, development, fruit ripening, and responses to biotic and abiotic stresses. Therefore, the understanding of the roles played by CuAOs and PAOs in these processes has progressed significantly during the recent decades, especially in rice and *Arabidopsis*. However, many key questions remain unanswered. Firstly, current studies show that the homeostasis regulation of PAs in plants is rather complex. So far, the information about specific regulatory mechanisms in PA biosynthesis and catabolism is very limited. Although it has been revealed, among others, that the transcription of PA biosynthetic genes is regulated by several transcription factors under stress (Paschalidis and Roubelakis-Angelakis, 2005a; Paschalidis et al., 2009a; Sun et al., 2014; Wu et al., 2016; Liu et al., 2018), relatively less information is available on the transcriptional regulation of PA catabolism. Secondly, although many members of CuAOs and PAOs involved in PA back-conversion pathway have been identified, the explicit role of the PA back-conversion reactions in PA homeostasis and associated physiological processes remains obscure. Last, but not least, although a dual signaling role for PA catabolism and the generated H_2_O_2_ under abiotic and/or biotic plant stress conditions has been revealed, further study will enable researchers to better elucidate this role by using new era technology.

## Author Contributions

WW, KP, J-CF, and JS wrote the paper. J-HL conceived the work and finalized the MS.

### Conflict of Interest Statement

The authors declare that the research was conducted in the absence of any commercial or financial relationships that could be construed as a potential conflict of interest.

## References

[ref1] Agudelo-Romero P. Bortolloti C. Pais M. S. Tiburcio A. F. Fortes A. M. (2013). Study of polyamines during grape ripening indicate an important role of polyamine catabolism. Plant Physiol. Biochem. 67, 105–119. 10.1016/j.plaphy.2013.02.024, PMID: 23562795

[ref2] Agurla S. Gayatri G. Raghavendra A. S. (2018). Polyamines increase nitric oxide and reactive oxygen species in guard cells of *Arabidopsis thaliana* during stomatal closure. Protoplasma 255, 153–162. 10.1007/s00709-017-1139-3, PMID: 28699025

[ref3] Ahmed S. Ariyaratne M. Patel J. Howard A. E. Kalinoski A. Phuntumart V.. (2017). Altered expression of polyamine transporters reveals a role for spermidine in the timing of flowering and other developmental response pathways. Plant Sci. 258, 146–155. 10.1016/j.plantsci.2016.12.002, PMID: 28330558

[ref4] Ahou A. Martignago D. Alabdallah O. Tavazza R. Stano P. Macone A.. (2014). A plant spermine oxidase/dehydrogenase regulated by the proteasome and polyamines. J. Exp. Bot. 65, 1585–1603. 10.1093/jxb/eru016, PMID: 24550437

[ref5] Alabdallah O. Ahou A. Mancuso N. Pompili V. Macone A. Pashkoulov D.. (2017). The Arabidopsis polyamine oxidase/dehydrogenase 5 interferes with cytokinin and auxin signaling pathways to control xylem differentiation. J. Exp. Bot. 68, 997–1012. 10.1093/jxb/erw510, PMID: 28199662

[ref6] Alcazar R. Marco F. Cuevas J. C. Patron M. Ferrando A. Carrasco P.. (2006). Involvement of polyamines in plant response to abiotic stress. Biotechnol. Lett. 28, 1867–1876. 10.1007/s10529-006-9179-3, PMID: 17028780

[ref7] Alcazar R. Planas J. Saxena T. Zarza X. Bortolotti C. Cuevas J.. (2010). Putrescine accumulation confers drought tolerance in transgenic Arabidopsis plants over-expressing the homologous Arginine decarboxylase 2 gene. Plant Physiol. Biochem. 48, 547–552. 10.1016/j.plaphy.2010.02.002, PMID: 20206537

[ref8] Andronis E. A. Moschou P. N. Roubelakis-Angelakis K. A. (2014). Peroxisomal polyamine oxidase and NADPH-oxidase cross-talk for ROS homeostasis which affects respiration rate in *Arabidopsis thaliana*. Front. Plant Sci. 5:132. 10.3389/fpls.2014.00132, PMID: 24765099 PMC3982065

[ref9] Andronis E. A. Roubelakis-Angelakis K. A. (2010). Short term salinity stress in tobacco plants leads to the onset of animal-like PCD hallmarks in planta in contrast to long term stress. Planta 231, 437–448. 10.1007/s00425-009-1060-x 19937341

[ref10] Angelini R. Cona A. Federico R. Fincato P. Tavladoraki P. Tisi A. (2010). Plant amine oxidases “on the move”: an update. Plant Physiol. Biochem. 48, 560–564. 10.1016/j.plaphy.2010.02.001, PMID: 20219383

[ref11] Angelini R. Tisi A. Rea G. Chen M. M. Botta M. Federico R.. (2008). Involvement of polyamine oxidase in wound healing. Plant Physiol. 146, 162–177. 10.4315/0362-028X-71.12.2488, PMID: 17993545 PMC2230557

[ref12] Biasi R. Bagni N. Costa G. (1988). Endogenous polyamines in apple and their relationship to fruitset and fruit growth. Plant Physiol. Biochem. 73, 201–205.

[ref13] Bleackley M. R. Wiltshire J. L. Perrine-Walker F. Vasa S. Burns R. L. Van Der Weerden N. L.. (2014). Agp2p, the plasma membrane transregulator of polyamine uptake, regulates the antifungal activities of the plant defensin NaD1 and other cationic peptides. Antimicrob. Agents Chemother. 58, 2688–2698. 10.1128/AAC.02087-13, PMID: 24566173 PMC3993230

[ref14] Bordenave C. D. Granados Mendoza C. Jimenez Bremont J. F. Garriz A. Rodriguez A. A. (2019). Defining novel plant polyamine oxidase subfamilies through molecular modeling and sequence analysis. BMC Evol. Biol. 19:28. 10.1186/s12862-019-1361-z 30665356 PMC6341606

[ref15] Cervelli M. Bianchi M. Cona A. Crosatti C. Stanca M. Angelini R.. (2006). Barley polyamine oxidase isoforms 1 and 2, a peculiar case of gene duplication. FEBS J. 273, 3990–4002. 10.1111/j.1742-4658.2006.05402.x, PMID: 16879612

[ref16] Cervelli M. Tavladoraki P. Agostino S. D. Angelini R. Federico R. Mariottini P. (2000). Isolation and characterization of three polyamine oxidase genes from *Zea mays*. Plant Physiol. Biochem. 38, 667–677. 10.1016/S0981-9428(00)01170-0

[ref17] Chen H. Cao Y. Li Y. Xia Z. Xie J. Carr J. P.. (2017). Identification of differentially regulated maize proteins conditioning Sugarcane mosaic virus systemic infection. New Phytol. 215, 1156–1172. 10.1111/nph.14645, PMID: 28627019

[ref18] Chen B. X. Li W. Y. Gao Y. T. Chen Z. J. Zhang W. N. Liu Q. J. (2016). Involvement of polyamine oxidase-produced hydrogen peroxide during coleorhiza-limited germination of rice seeds. Front. Plant Sci. 7:1219. 10.3389/fpls.2016.01219 27570530 PMC4981591

[ref19] Chen T. Li W. Hu X. Guo J. Liu A. Zhang B. (2015). A cotton MYB transcription factor, GbMYB5, is positively involved in plant adaptive response to drought stress. Plant Cell Physiol. 56, 917–929. 10.1093/pcp/pcv019, PMID: 25657343

[ref20] Cohen S. Balint R. Sindhu R. K. Marcu D. (1981). Polyamine biosynthesis and metabolism in normal and virus-infected plant protoplasts. Med. Biol. 59, 394–402. PMID: 7339302

[ref21] Cona A. Rea G. Angelini R. Federico R. Tavladoraki P. (2006). Functions of amine oxidases in plant development and defence. Trends Plant Sci. 11, 80–88. 10.1016/j.tplants.2005.12.009, PMID: 16406305

[ref22] Corpas F. J. Del Rio L. A. Palma J. M. (2019). Plant peroxisomes at the crossroad of NO and H_2_O_2_ metabolism. J. Integr. Plant Biol. 10.1111/jipb.12772, PMID: 30609289

[ref23] Cowley T. Walters D. R. (2002). Polyamine metabolism in barley reacting hypersensitively to the powdery mildew fungus Blumeria graminis f. sp. hordei. Plant Cell Environ. 25, 461–468. 10.1046/j.0016-8025.2001.00819.x

[ref24] Dorighetto Cogo A. J. Dutra Ferreira K. D. R. Okorokov L. A. Ramos A. C. Facanha A. R. Okorokova-Facanha A. L. (2018). Spermine modulates fungal morphogenesis and activates plasma membrane H^+^-ATPase during yeast to hyphae transition. Biol. Open 7, 1–13. 10.1242/bio.029660, PMID: 29361612 PMC5861359

[ref25] Estiarte N. Crespo-Sempere A. Marin S. Sanchis V. Ramos A. J. (2017). Exploring polyamine metabolism of Alternaria alternata to target new substances to control the fungal infection. Food Microbiol. 65, 193–204. 10.1016/j.fm.2017.02.001, PMID: 28400003

[ref26] Fincato P. Moschou P. N. Ahou A. Angelini R. Roubelakis-Angelakis K. A. Federico R.. (2012). The members of *Arabidopsis thaliana* PAO gene family exhibit distinct tissue- and organ-specific expression pattern during seedling growth and flower development. Amino Acids 42, 831–841. 10.1007/s00726-011-0999-7, PMID: 21814784

[ref27] Fincato P. Moschou P. N. Spedaletti V. Tavazza R. Angelini R. Federico R.. (2011). Functional diversity inside the Arabidopsis polyamine oxidase gene family. J. Exp. Bot. 62, 1155–1168. 10.1093/jxb/erq341, PMID: 21081665

[ref28] Fortes A. M. Agudelo-Romero P. (2018). Polyamine metabolism in climacteric and non-climacteric fruit ripening. Methods Mol. Biol. 1694, 433–447. 10.1007/978-1-4939-7398-9_36 29080186

[ref29] Fortes A. M. Teixeira R. T. Agudelo-Romero P. (2015). Complex interplay of hormonal signals during grape berry ripening. Molecules 20, 9326–9343. 10.3390/molecules20059326, PMID: 26007186 PMC6272489

[ref30] Garriz A. Dalmasso M. C. Pieckenstain F. L. Ruiz O. A. (2003). The putrescine analogue 1-aminooxy-3-aminopropane perturbs polyamine metabolism in the phytopathogenic fungus *Sclerotinia sclerotiorum*. Arch. Microbiol. 180, 169–175. 10.1007/s00203-003-0572-1, PMID: 12851744

[ref31] Gemes K. Kim Y. J. Park K. Y. Moschou P. N. Andronis E. Valassaki C.. (2016). An NADPH-oxidase/polyamine oxidase feedback loop controls oxidative burst under salinity. Plant Physiol. 172, 1418–1431. 10.1104/pp.16.01118, PMID: 27600815 PMC5100782

[ref32] Gemes K. Mellidou I. Karamanoli K. Beris D. Park K. Y. Matsi T.. (2017). Deregulation of apoplastic polyamine oxidase affects development and salt response of tobacco plants. J. Plant Physiol. 211, 1–12. 10.1016/j.jplph.2016.12.012, PMID: 28135604

[ref33] Ghuge S. A. Tisi A. Carucci A. Rodrigues-Pousada R. A. Franchi S. Tavladoraki P.. (2015). Cell wall amine oxidases: new players in root xylem differentiation under stress conditions. Plants 4, 489–504. 10.3390/plants4030489, PMID: 27135338 PMC4844406

[ref34] Gomez-Jimenez M. C. Paredes M. A. Gallardo M. Fernandez-Garcia N. Olmos E. Sanchez-Calle I. M. (2010). Tissue-specific expression of olive S-adenosyl methionine decarboxylase and spermidine synthase genes and polyamine metabolism during flower opening and early fruit development. Planta 232, 629–647. 10.1007/s00425-010-1198-6, PMID: 20532909

[ref35] Groβ F. Rudolf E. E. Thiele B. Durner J. Astier J. (2017). Copper amine oxidase 8 regulates arginine-dependent nitric oxide production in *Arabidopsis thaliana*. J. Exp. Bot. 68, 2149–2162. 10.1093/jxb/erx105 28383668 PMC5447880

[ref36] Guo J. Wang S. Yu X. Dong R. Li Y. Mei X.. (2018). Polyamines regulate strawberry fruit ripening by abscisic acid, auxin, and ethylene. Plant Physiol. 177, 339–351. 10.1104/pp.18.00245, PMID: 29523717 PMC5933135

[ref37] Gupta K. J. Brotman Y. Segu S. Zeier T. Zeier J. Persijn S. T.. (2013). The form of nitrogen nutrition affects resistance against *Pseudomonas syringae* pv. phaseolicola in tobacco. J. Exp. Bot. 64, 553–568. 10.1093/jxb/ers348, PMID: 23230025 PMC3542047

[ref38] Gupta K. Sengupta A. Chakraborty M. Gupta B. (2016). Hydrogen peroxide and polyamines act as double edged swords in plant abiotic stress responses. Front. Plant Sci. 7, 1–19. 10.3389/fpls.2016.01343 27672389 PMC5018498

[ref39] Handa A. K. Fatima T. Mattoo A. K. (2018). Polyamines: bio-molecules with diverse functions in plant and human health and disease. Front. Chem. 6:10. 10.3389/fchem.2018.00010 29468148 PMC5807879

[ref40] Hao Y. Huang B. Jia D. Mann T. Jiang X. Qiu Y.. (2018). Identification of seven polyamine oxidase genes in tomato (*Solanum lycopersicum* L.) and their expression profiles under physiological and various stress conditions. J. Plant Physiol. 228, 1–11. 10.1016/j.jplph.2018.05.004, PMID: 29793152

[ref41] Hatmi S. Villaume S. Trotel-Aziz P. Barka E. A. Clement C. Aziz A. (2018). Osmotic stress and ABA affect immune response and susceptibility of grapevine berries to gray mold by priming polyamine accumulation. Front. Plant Sci. 9:1010. 10.3389/fpls.2018.01010 30050554 PMC6050403

[ref42] Ioannidis N. E. Malliarakis D. Torne J. M. Santos M. Kotzabasis K. (2016). The over-expression of the plastidial transglutaminase from maize in arabidopsis increases the activation threshold of photoprotection. Front. Plant Sci. 7:635. 10.3389/fpls.2016.00635, PMID: 27242838 PMC4861818

[ref43] Ioannidis N. E. Sfichi L. Kotzabasis K. (2006). Putrescine stimulates chemiosmotic ATP synthesis. BBA-Bioenergetics 1757, 821–828. 10.1371/journal.pone.0000005, PMID: 16828052

[ref44] Kamada-Nobusada T. Hayashi M. Fukazawa M. Sakakibara H. Nishimura M. (2008). A putative peroxisomal polyamine oxidase, AtPAO4, is involved in polyamine catabolism in *Arabidopsis thaliana*. Plant Cell Physiol. 49, 1272–1282. 10.1093/pcp/pcn114, PMID: 18703589

[ref45] Kim D. W. Watanabe K. Murayama C. Izawa S. Niitsu M. Michael A. J.. (2014). Polyamine oxidase5 regulates arabidopsis growth through thermospermine oxidase activity. Plant Physiol. 165, 1575–1590. 10.1104/pp.114.242610, PMID: 24906355 PMC4119040

[ref46] Kitashiba H. Honda C. Moriguchi T. (2006). Identification of polyamine oxidase genes from apple and expression analysis during fruit development and cell growth. Plant Biotechnol. 23, 425–429. 10.5511/plantbiotechnology.23.425

[ref47] Koç E. (2015). Exogenous application of spermidine enhanced tolerance of pepper against *phytophthora capsici* stress. Plant Prot. Sci. 51, 127–135. 10.17221/86/2014-PPS

[ref48] Kusano T. Berberich T. Tateda C. Takahashi Y. (2008). Polyamines: essential factors for growth and survival. Planta 228, 367–381. 10.1007/s00425-008-0772-7, PMID: 18594857

[ref49] Kushad M. M. Yelenosky G. Knight R. (1988). Interrelationship of polyamine and ethylene biosynthesis during avocado fruit development and ripening. Plant Physiol. Biochem. 87, 463–467. 10.1104/pp.87.2.463 PMC1054775 16666165

[ref50] Liu T. Dobashi H. Kim D. W. Sagor G. H. Niitsu M. Berberich T. (2014a). Arabidopsis mutant plants with diverse defects in polyamine metabolism show unequal sensitivity to exogenous cadaverine probably based on their spermine content. Physiol. Mol. Biol. Plants 20, 151–159. 10.1007/s12298-014-0227-5 24757319 PMC3988325

[ref51] Liu T. Huang B. Chen L. Xian Z. Song S. Chen R.. (2018). Genome-wide identification, phylogenetic analysis, and expression profiling of polyamine synthesis gene family members in tomato. Gene 661, 1–10. 10.1016/j.gene.2018.03.084, PMID: 29605609

[ref52] Liu T. Kim D. W. Niitsu M. Berberich T. Kusano T. (2014b). *Oryza sativa* polyamine oxidase 1 back-converts tetraamines, spermine and thermospermine, to spermidine. Plant Cell Rep. 33, 143–151. 10.1007/s00299-013-1518-y 24105034

[ref53] Liu T. Kim D. W. Niitsu M. Maeda S. Watanabe M. Kamio Y. (2014c). Polyamine oxidase 7 is a terminal catabolism-type enzyme in *Oryza sativa* and is specifically expressed in anthers. Plant Cell Physiol. 55, 1110–1122. 10.1093/pcp/pcu047 24634478

[ref54] Liu J. H. Moriguchi T. (2007). Changes in free polyamine titers and expression of polyamine biosynthetic genes during growth of peach in vitro callus. Plant Cell Rep. 26, 125–131. 10.1007/s00299-006-0223-5, PMID: 16912865

[ref55] Liu J. H. Nada K. Honda C. Kitashiba H. Wen X. P. Pang X. M.. (2006). Polyamine biosynthesis of apple callus under salt stress: importance of the arginine decarboxylase pathway in stress response. J. Exp. Bot. 57, 2589–2599. 10.1093/jxb/erl018, PMID: 16825316

[ref56] Liu J.-H. Wang W. Wu H. Gong X. Moriguchi T. (2015). Polyamines function in stress tolerance: from synthesis to regulation. Front. Plant Sci. 6, 1–10. 10.3389/fpls.2015.00827 26528300 PMC4602114

[ref57] Liu T. Wook Kim D. Niitsu M. Berberich T. Kusano T. (2014d). POLYAMINE OXIDASE 1 from rice (*Oryza sativa*) is a functional ortholog of Arabidopsis POLYAMINE OXIDASE 5. Plant Signal. Behav. 9:e29773. 10.4161/psb.29773 25763711 PMC4205151

[ref58] Lowe-Power T. M. Hendrich C. G. Von Roepenack-Lahaye E. Li B. Wu D. Mitra R.. (2018). Metabolomics of tomato xylem sap during bacterial wilt reveals *Ralstonia solanacearum* produces abundant putrescine, a metabolite that accelerates wilt disease. Environ. Microbiol. 20, 1330–1349. 10.1111/1462-2920.14020, PMID: 29215193 PMC5903990

[ref59] Majumdar R. Barchi B. Turlapati S. A. Gagne M. Minocha R. Long S.. (2016). Glutamate, ornithine, arginine, proline, and polyamine metabolic interactions: the pathway is regulated at the post-transcriptional level. Front. Plant Sci. 7:78. 10.3389/fpls.2016.00078, PMID: 26909083 PMC4754450

[ref60] Malik A. U. Singh Z. (2004). Endogenous free polyamines of mangos in relation to development and ripening. J. Am. Soc. Hortic. Sci. 129, 280–286. 10.21273/JASHS.129.3.0280

[ref61] Marina M. Maiale S. J. Rossi F. R. Romero M. F. Rivas E. I. Garriz A.. (2008). Apoplastic polyamine oxidation plays different roles in local responses of tobacco to infection by the necrotrophic fungus *Sclerotinia sclerotiorum* and the biotrophic bacterium *Pseudomonas viridiflava*. Plant Physiol. 147, 2164–2178. 10.1104/pp.108.122614, PMID: 18583531 PMC2492638

[ref62] Marina M. Sirera F. V. Rambla J. L. Gonzalez M. E. Blazquez M. A. Carbonell J.. (2013). Thermospermine catabolism increases *Arabidopsis thaliana* resistance to *Pseudomonas viridiflava*. J. Exp. Bot. 64, 1393–1402. 10.1093/jxb/ert012, PMID: 23382552

[ref63] Marini F. Betti L. Scramagli S. Biondi S. Torrigiani P. (2001). Polyamine metabolism is upregulated in response to tobacco mosaic virus in hypersensitive, but not in susceptible, tobacco. New Phytol. 149, 301–309. 10.1046/j.1469-8137.2001.00017.x 33874627

[ref64] Mattoo A. K. Chung S. H. Goyal R. K. Fatima T. Solomos T. Srivastava A.. (2007). Overaccumulation of higher polyamines in ripening transgenic tomato fruit revives metabolic memory, upregulates anabolism-related genes, and positively impacts nutritional quality. J. AOAC Int. 90, 1456–1464. PMID: 17955994

[ref65] Mellidou I. Karamanoli K. Beris D. Haralampidis K. Constantinidou H. A. Roubelakis-Angelakis K. A. (2017). Underexpression of apoplastic polyamine oxidase improves thermotolerance in *Nicotiana tabacum*. J. Plant Physiol. 218, 171–174. 10.1016/j.jplph.2017.08.006, PMID: 28886452

[ref66] Mellidou I. Moschou P. N. Ioannidis N. Pankou C. Gémes K. Valassakis C. (2016). Silencing S-Adenosyl-L-Methionine Decarboxylase (SAMDC) in Nicotiana tabacum Points at a Polyamine-Dependent Trade-Off between Growth and Tolerance Responses. Front. Plant Sci. 7, 1–17. 10.3389/fpls.2016.00379 27064210 PMC4814703

[ref67] Møller S. G. Mcpherson M. J. (1998). Developmental expression and biochemical analysis of the Arabidopsis atao1 gene encoding an H_2_O_2_-generating diamine oxidase. Plant J. 13, 781–791. 9681017 10.1046/j.1365-313x.1998.00080.x

[ref68] Montilla-Bascon G. Rubiales D. Hebelstrup K. H. Mandon J. Harren F. J. M. Cristescu S. M. (2017). Reduced nitric oxide levels during drought stress promote drought tolerance in barley and is associated with elevated polyamine biosynthesis. Sci. Rep. 7:13311. 10.1038/s41598-017-13458-1 29042616 PMC5645388

[ref69] Moschou P. N. Delis I. D. Paschalidis K. A. Roubelakis-Angelakis K. A. (2008a). Transgenic tobacco plants overexpressing polyamine oxidase are not able to cope with oxidative burst generated by abiotic factors. Physiol. Plant. 133, 140–156. 10.1111/j.1399-3054.2008.01049.x 18282192

[ref70] Moschou P. N. Paschalidis K. A. Delis I. D. Andriopoulou A. H. Lagiotis G. D. Yakoumakis D. I. (2008b). Spermidine exodus and oxidation in the apoplast induced by abiotic stress is responsible for H_2_O_2_ signatures that direct tolerance responses in tobacco. Plant Cell 20, 1708–1724. 10.1105/tpc.108.059733 18577660 PMC2483379

[ref71] Moschou P. N. Paschalidis K. A. Roubelakis-Angelakis K. A. (2008c). Plant polyamine catabolism: The state of the art. Plant Signal. Behav. 3, 1061–1066. 10.4161/psb.3.12.7172 19513239 PMC2634460

[ref72] Moschou P. N. Roubelakis-Angelakis K. A. (2014). Polyamines and programmed cell death. J. Exp. Bot. 65, 1285–1296. 10.1093/jxb/ert373, PMID: 24218329

[ref73] Moschou P. N. Sanmartin M. Andriopoulou A. H. Rojo E. Sanchez-Serrano J. J. Roubelakis-Angelakis K. A. (2009b). Bridging the gap between plant and mammalian polyamine catabolism: a novel peroxisomal polyamine oxidase responsible for a full back-conversion pathway in Arabidopsis. Plant Physiol. 147, 1845–1857. 10.1104/pp.108.123802 PMC2492618 18583528

[ref74] Moschou P. N. Sarris P. F. Skandalis N. Andriopoulou A. H. Paschalidis K. A. Panopoulos N. J. (2009a). Engineered polyamine catabolism preinduces tolerance of tobacco to bacteria and oomycetes. Plant Physiol. 149, 1970–1981. 10.1104/pp.108.134932 19218362 PMC2663742

[ref75] Moschou P. N. Wu J. Cona A. Tavladoraki P. Angelini R. Roubelakis-Angelakis K. A. (2012). The polyamines and their catabolic products are significant players in the turnover of nitrogenous molecules in plants. J. Exp. Bot. 63, 5003–5015. 10.1093/jxb/ers202, PMID: 22936828

[ref76] Naconsie M. Kato K. Shoji T. Hashimoto T. (2014). Molecular evolution of N-methylputrescine oxidase in tobacco. Plant Cell Physiol. 55, 436–444. 10.1093/pcp/pct179, PMID: 24287136

[ref77] Negrel J. Vallee J. C. Martin C. (1984). Ornithine decarboxylase activity and the hypersensitive reaction of tobacco to tobacco mosaic virus in *Nicotiana tabacum*. Phytochemistry 23, 2747–2751. 10.1016/0031-9422(84)83008-3

[ref78] Nguyen H. C. Lin K. H. Ho S. L. Chiang C. M. Yang C. M. (2018). Enhancing the abiotic stress tolerance of plants: from chemical treatment to biotechnological approaches. Physiol. Plant. 164, 452–466. 10.1111/ppl.12812, PMID: 30054915

[ref79] Ono Y. Kim D. W. Watanabe K. Sasaki A. Niitsu M. Berberich T.. (2012). Constitutively and highly expressed *Oryza sativa* polyamine oxidases localize in peroxisomes and catalyze polyamine back conversion. Amino Acids 42, 867–876. 10.1007/s00726-011-1002-3, PMID: 21796433

[ref80] Pal M. Ivanovska B. Olah T. Tajti J. Hamow K. A. Szalai G.. (2019). Role of polyamines in plant growth regulation of Rht wheat mutants. Plant Physiol. Biochem. 137, 189–202. 10.1016/j.plaphy.2019.02.013, PMID: 30798173

[ref81] Papadakis A. K. Paschalidis K. A. Roubelakis-Angelakis K. A. (2005). Biosynthesis profile and endogenous titers of polyamines differ in totipotent and recalcitrant plant protoplasts. Physiol. Plant. 125, 10–20. 10.1111/j.1399-3054.2005.00550.x

[ref82] Papadakis A. K. Roubelakis-Angelakis K. A. (2005). Polyamines inhibit NADPH oxidase-mediated superoxide generation and putrescine prevents programmed cell death induced by polyamine oxidase-generated hydrogen peroxide. Planta 220, 826–837. 10.1007/s00425-004-1400-9, PMID: 15517351

[ref83] Paschalidis K. Moschou P. N. Aziz A. Toumi I. Roubelakis-Angelakis Κ. Α. (2009a). “Polyamines in grapevine: an update” in Grapevine molecular physiology & biotechnology. ed. Roubelakis-Angelakis K. A. (Switzerland AG: Springer Netherlands Publishers). 10.1007/978-90-481-2305-6_8

[ref84] Paschalidis K. A. Moschou P. N. Toumi I. Roubelakis-Angelakis K. A. (2009b). Polyamine anabolic/catabolic regulation along the woody grapevine plant axis. J. Plant Physiol. 166, 1508–1519. 10.1016/j.jplph.2009.03.013 19450900

[ref85] Paschalidis K. A. Roubelakis-Angelakis K. A. (2005a). Spatial and temporal distribution of polyamine levels and polyamine anabolism in different organs/tissues of the tobacco plant. Correlations with age, cell division/expansion, and differentiation. Plant Physiol. 138, 142–152. 10.1104/pp.104.055483 15849310 PMC1104170

[ref86] Paschalidis K. A. Roubelakis-Angelakis K. A. (2005b). Sites and regulation of polyamine catabolism in the tobacco plant. Correlations with cell division/expansion, cell cycle progression, and vascular development. Plant Physiol. 138, 2174–2184. 10.1104/pp.105.063941 16040649 PMC1183405

[ref87] Paschalidis K. A. Toumi I. Moschou P. N. Roubelakis-Angelakis K. A. (2010). ABA-dependent amine oxidases-derived H_2_O_2_ affects stomata conductance. Plant Signal. Behav. 5, 1153–1156. 10.4161/psb.5.9.12679 21490422 PMC3115092

[ref88] Pegg A. E. (2014). The function of spermine. IUBMB Life 66, 8–18. 10.1002/iub.1237, PMID: 24395705

[ref89] Planas-Portell J. Gallart M. Tiburcio A. F. Altabella T. (2013). Copper-containing amine oxidases contribute to terminal polyamine oxidation in peroxisomes and apoplast of *Arabidopsis thaliana*. BMC Plant Biol. 13:109. 10.1186/1471-2229-13-109, PMID: 23915037 PMC3751259

[ref90] Podlesakova K. Ugena L. Spichal L. Dolezal K. De Diego N. (2019). Phytohormones and polyamines regulate plant stress responses by altering GABA pathway. New Biotechnol. 48, 53–65. 10.1016/j.nbt.2018.07.003, PMID: 30048769

[ref91] Pottosin I. Velarde-Buendia A. M. Bose J. Fuglsang A. T. Shabala S. (2014a). Polyamines cause plasma membrane depolarization, activate Ca2^+^-, and modulate H^+^-ATPase pump activity in pea roots. J. Exp. Bot. 65, 2463–2472. 10.1093/jxb/eru133 24723394

[ref92] Pottosin I. Velarde-Buendia A. M. Bose J. Zepeda-Jazo I. Shabala S. Dobrovinskaya O. (2014b). Cross-talk between reactive oxygen species and polyamines in regulation of ion transport across the plasma membrane: implications for plant adaptive responses. J. Exp. Bot. 65, 1271–1283. 10.1093/jxb/ert423 24465010

[ref93] Rangan P. Subramani R. Kumar R. Singh A. K. Singh R. (2014). Recent advances in polyamine metabolism and abiotic stress tolerance. Biomed. Res. Int. 2014:239621. 10.1155/2014/239621, PMID: 25136565 PMC4124767

[ref94] Rastogi R. Davies P. J. (1991). Polyamine metabolism in ripening tomato fruit: II. Polyamine metabolism and synthesis in relation to enhanced putrescine content and storage life of a/c tomato fruit. Plant Physiol. 95, 41–45. 10.1104/pp.95.1.41, PMID: 16667978 PMC1077482

[ref95] Rea G. Laurenzi M. Tranquilli E. D’ovidio R. Federico R. Angelini R. (1998). Developmentally and wound-regulated expression of the gene encoding a cell wall copper amine oxidase in chickpea seedlings. FEBS Lett. 437, 177–182. 10.1016/S0014-5793(98)01219-8, PMID: 9824285

[ref96] Recalde L. Vazquez A. Groppa M. D. Benavides M. P. (2018). Reactive oxygen species and nitric oxide are involved in polyamine-induced growth inhibition in wheat plants. Protoplasma 255, 1295–1307. 10.1007/s00709-018-1227-z, PMID: 29511833

[ref97] Rentel M. C. Lecourieux D. Ouaked F. (2004). OXI1 kinase is necessary for oxidative burst-mediated signalling in Arabidopsis. Nat. Rev. Genet. 427, 858–861. 10.1038/nature02353 14985766

[ref98] Rodriguez A. A. Maiale S. J. Menendez A. B. Ruiz O. A. (2009). Polyamine oxidase activity contributes to sustain maize leaf elongation under saline stress. J. Exp. Bot. 60, 4249–4262. 10.1093/jxb/erp256, PMID: 19717530

[ref99] Rossi F. R. Marina M. Pieckenstain F. L. (2015). Role of Arginine decarboxylase (ADC) in *Arabidopsis thaliana* defence against the pathogenic bacterium *Pseudomonas viridiflava*. Plant Biol. 17, 831–839. 10.1111/plb.12289, PMID: 25409942

[ref100] Rossi F. R. Romero F. M. Ruiz O. A. Marina M. Garriz A. (2018). Phenotypic and genotypic characterization of mutant plants in polyamine metabolism genes during pathogenic interactions. Methods Mol. Biol. 1694, 405–416. 10.1007/978-1-4939-7398-9_33 29080183

[ref101] Sagor G. H. M. Kusano T. Berberich T. (2017). Identification of the actual coding region for polyamine oxidase 6 from rice (OsPAO6) and its partial characterization. Plant Signal. Behav. 12:e1359456. 10.1080/15592324.2017.1359456, PMID: 28786735 PMC5616144

[ref102] Sayas E. Perez-Benavente B. Manzano C. Farras R. Alejandro S. Del Pozo J. C.. (2019). Polyamines interfere with protein ubiquitylation and cause depletion of intracellular amino acids: a possible mechanism for cell growth inhibition. FEBS Lett. 593, 209–218. 10.1002/1873-3468.13299, PMID: 30447065

[ref103] Sebela M. Radova A. Angelini R. Tavladoraki P. Frebort I. I. Pec P. (2001). FAD-containing polyamine oxidases: a timely challenge for researchers in biochemistry and physiology of plants. Plant Sci. 160, 197–207. 10.1016/S0168-9452(00)00380-0, PMID: 11164591

[ref104] Shabala S. Bose J. Fuglsang A. T. Pottosin I. (2016). On a quest for stress tolerance genes: membrane transporters in sensing and adapting to hostile soils. J. Exp. Bot. 67, 1015–1031. 10.1093/jxb/erv465, PMID: 26507891

[ref105] Simpson C. G. Cullen D. W. Hackett C. A. Smith K. Hallett P. D. Mcnicol J.. (2017). Mapping and expression of genes associated with raspberry fruit ripening and softening. Theor. Appl. Genet. 130, 557–572. 10.1007/s00122-016-2835-7, PMID: 27942774

[ref106] Skopelitis D. S. Paranychianakis N. V. Pliakonis E. D. Paschalidis K. A. Delis I. D. Yakoumakis D. I.. (2006). Abiotic stress generates ROS that signal expression of anionic glutamate dehydrogenases to form glutamate for proline synthesis in tobacco and grapevine. Plant Cell 18, 2767–2781. 10.1105/tpc.105.038323, PMID: 17041150 PMC1626620

[ref107] Smith M. A. Davies P. J. (1985). Separation and quantitation of polyamines in plant tissue by high performance liquid chromatography of their dansyl derivatives. Plant Physiol. 78, 89–91. 10.1104/pp.78.1.89, PMID: 16664216 PMC1064682

[ref108] Sobieszczuk-Nowicka E. (2017). Polyamine catabolism adds fuel to leaf senescence. Amino Acids 49, 49–56. 10.1007/s00726-016-2377-y, PMID: 28039518 PMC5241338

[ref109] Sobieszczuk-Nowicka E. Kubala S. Zmienko A. Malecka A. Legocka J. (2015). From accumulation to degradation: reprogramming polyamine metabolism facilitates dark-induced senescence in barley leaf cells. Front. Plant Sci. 6:1198. 10.3389/fpls.2015.01198, PMID: 26779231 PMC4702279

[ref110] Stes E. Biondi S. Holsters M. Vereecke D. (2011). Bacterial and plant signal integration via D3-type cyclins enhances symptom development in the Arabidopsis-Rhodococcus *fascians interaction*. Plant Physiol. 156, 712–725. 10.1104/pp.110.171561, PMID: 21459976 PMC3177270

[ref111] Su G. X. Yu B. J. Zhang W. H. Liu Y. L. (2007). Higher accumulation of γ-aminobutyric acid induced by salt stress through stimulating the activity of diamine oxidases in *Glycine max* (L.) Merr. roots. Plant Physiol. Biochem. 45, 560–566. 10.1016/j.plaphy.2007.05.007 17624796

[ref112] Sun P. Zhu X. Huang X. Liu J. H. (2014). Overexpression of a stress-responsive MYB transcription factor of *Poncirus trifoliata* confers enhanced dehydration tolerance and increases polyamine biosynthesis. Plant Physiol. Biochem. 78, 71–79. 10.1016/j.plaphy.2014.02.022, PMID: 24636909

[ref113] Takacs Z. Poor P. Tari I. (2016). Comparison of polyamine metabolism in tomato plants exposed to different concentrations of salicylic acid under light or dark conditions. Plant Physiol. Biochem. 108, 266–278. 10.1016/j.plaphy.2016.07.020, PMID: 27474934

[ref114] Takahashi Y. Cong R. Sagor G. H. Niitsu M. Berberich T. Kusano T. (2010). Characterization of five polyamine oxidase isoforms in *Arabidopsis thaliana*. Plant Cell Rep. 29, 955–965. 10.1007/s00299-010-0881-1, PMID: 20532512

[ref115] Takahashi T. Kakehi J. (2010). Polyamines: ubiquitous polycations with unique roles in growth and stress responses. Ann. Bot. 105, 1–6. 10.1093/aob/mcp259, PMID: 19828463 PMC2794062

[ref116] Takahashi Y. Ono K. Akamine Y. Asano T. Ezaki M. Mouri I. (2018). Highly-expressed polyamine oxidases catalyze polyamine back conversion in *Brachypodium distachyon*. J. Plant Res. 131, 341–348. 10.1007/s10265-017-0989-2, PMID: 29063977

[ref117] Tanou G. Ziogas V. Belghazi M. Christou A. Filippou P. Job D.. (2014). Polyamines reprogram oxidative and nitrosative status and the proteome of citrus plants exposed to salinity stress. Plant Cell Environ. 37, 864–885. 10.1111/pce.12204, PMID: 24112028

[ref118] Tassoni A. Watkins C. B. Davies P. J. (2006). Inhibition of the ethylene response by 1-MCP in tomato suggests that polyamines are not involved in delaying ripening, but may moderate the rate of ripening or over-ripening. J. Exp. Bot. 57, 3313–3325. 10.1093/jxb/erl092, PMID: 16920766

[ref119] Tavladoraki P. Cona A. Angelini R. (2016). Copper-containing amine oxidases and FAD-dependent polyamine oxidases are key players in plant tissue differentiation and organ development. Front. Plant Sci. 7:824. 10.3389/fpls.2016.00824, PMID: 27446096 PMC4923165

[ref120] Tavladoraki P. Rossi M. N. Saccuti G. Perez-Amador M. A. Polticelli F. Angelini R.. (2006). Heterologous expression and biochemical characterization of a polyamine oxidase from Arabidopsis involved in polyamine back conversion. Plant Physiol. 141, 1519–1532. 10.1104/pp.106.080911, PMID: 16778015 PMC1533960

[ref121] Tavladoraki P. Schinina M. E. Cecconi F. Di Agostino S. Manera F. Rea G.. (1998). Maize polyamine oxidase: primary structure from protein and cDNA sequencing. FEBS Lett. 426, 62–66. 10.1016/S0014-5793(98)00311-1, PMID: 9598979

[ref122] Teh H. F. Neoh B. K. Wong Y. C. Kwong Q. B. Ooi T. E. Ng T. L.. (2014). Hormones, polyamines, and cell wall metabolism during oil palm fruit mesocarp development and ripening. J. Agric. Food Chem. 62, 8143–8152. 10.1021/jf500975h, PMID: 25032485

[ref123] Tiburcio A. F. Alcazar R. (2018). Potential applications of polyamines in agriculture and plant biotechnology. Methods Mol. Biol. 1694, 489–508. 10.1007/978-1-4939-7398-9_40 29080190

[ref124] Tipping A. J. Mcpherson M. J. (1995). Cloning and molecular analysis of the pea seedling copper amine oxidase. J. Biol. Chem. 270, 16939–16946. 10.1074/jbc.270.28.16939, PMID: 7622512

[ref125] Tisi A. Federico R. Moreno S. Lucretti S. Moschou P. N. Roubelakis-Angelakis K. A.. (2011). Perturbation of polyamine catabolism can strongly affect root development and xylem differentiation. Plant Physiol. 157, 200–215. 10.1104/pp.111.173153, PMID: 21746808 PMC3165870

[ref126] Toumi I. Moschou P. N. Paschalidis K. A. Bouamama B. Ben Salem-Fnayou A. Ghorbel A. W.. (2010). Abscisic acid signals reorientation of polyamine metabolism to orchestrate stress responses via the polyamine exodus pathway in grapevine. J. Plant Physiol. 167, 519–525. 10.1016/j.jplph.2009.10.022, PMID: 20060616

[ref127] Tsaniklidis G. Kotsiras A. Tsafouros A. Roussos P. A. Aivalakis G. Katinakis P.. (2016). Spatial and temporal distribution of genes involved in polyamine metabolism during tomato fruit development. Plant Physiol. Biochem. 100, 27–36. 10.1016/j.plaphy.2016.01.001, PMID: 26773542

[ref128] Tuskan G. A. Difazio S. Jansson S. Bohlmann J. Grigoriev I. Hellsten U.. (2006). The genome of black cottonwood, *Populus trichocarpa* (Torr. & Gray). Science 313, 1596–1604. 10.1126/science.1128691, PMID: 16973872

[ref129] Valdes-Santiago L. Cervantes-Chavez J. A. Leon-Ramirez C. G. Ruiz-Herrera J. (2012). Polyamine metabolism in fungi with emphasis on phytopathogenic species. J. Amino Acids 2012:837932. 10.1155/2012/837932, PMID: 22957208 PMC3432380

[ref130] Van de Poel B. Bulens I. Oppermann Y. Hertog M. L. Nicolai B. M. Sauter M.. (2013). S-adenosyl-L-methionine usage during climacteric ripening of tomato in relation to ethylene and polyamine biosynthesis and transmethylation capacity. Physiol. Plant. 148, 176–188. 10.1111/j.1399-3054.2012.01703.x, PMID: 23020643

[ref131] Vilas J. M. Romero F. M. Rossi F. R. Marina M. Maiale S. J. Calzadilla P. I.. (2018). Modulation of plant and bacterial polyamine metabolism during the compatible interaction between tomato and *Pseudomonas syringae*. J. Plant Physiol. 231, 281–290. 10.1016/j.jplph.2018.09.014, PMID: 30342327

[ref132] Wang D. Li L. Xu Y. Limwachiranon J. Li D. Ban Z.. (2017). Effect of exogenous nitro oxide on chilling tolerance, polyamine, proline, and gamma-aminobutyric acid in bamboo shoots (*Phyllostachys praecox* f. prevernalis). J. Agric. Food Chem. 65, 5607–5613. 10.1021/acs.jafc.7b02091, PMID: 28648058

[ref133] Wang W. Liu J. H. (2015). Genome-wide identification and expression analysis of the polyamine oxidase gene family in sweet orange (*Citrus sinensis*). Gene 555, 421–429. 10.1016/j.gene.2014.11.042, PMID: 25445392

[ref134] Wang W. Liu J. H. (2016). CsPAO4 of *Citrus sinensis* functions in polyamine terminal catabolism and inhibits plant growth under salt stress. Sci. Rep. 6:31384. 10.1038/srep31384 27535697 PMC4989168

[ref135] Wang H. Liu B. Li H. Zhang S. (2016). Identification and biochemical characterization of polyamine oxidases in amphioxus: implications for emergence of vertebrate-specific spermine and acetylpolyamine oxidases. Gene 575, 429–437. 10.1016/j.gene.2015.09.017, PMID: 26367330

[ref136] Wang Y. Ye X. Yang K. Shi Z. Wang N. Yang L.. (2019). Characterization, expression, and functional analysis of polyamine oxidases for their role in selenium-induced hydrogen peroxide production in *Brassica rapa*. J. Sci. Food Agric. 10.1002/jsfa.9638, PMID: 30761554

[ref137] Waszczak C. Carmody M. Kangasjarvi J. (2018). Reactive oxygen species in plant signaling. Annu. Rev. Plant Biol. 69, 209–236. 10.1146/annurev-arplant-042817-040322, PMID: 29489394

[ref138] Wojtasik W. Kulma A. Namysl K. Preisner M. Szopa J. (2015). Polyamine metabolism in flax in response to treatment with pathogenic and non-pathogenic *Fusarium strains*. Front. Plant Sci. 6:291. 10.3389/fpls.2015.00291, PMID: 25972886 PMC4413726

[ref139] Wu H. Fu B. Sun P. Xiao C. Liu J. H. (2016). A NAC transcription factor represses putrescine biosynthesis and affects drought tolerance. Plant Physiol. 172, 1532–1547. 10.1104/pp.16.01096, PMID: 27663409 PMC5100760

[ref140] Wu J. Qu H. Shang Z. Jiang X. Moschou P. N. Roubelakis-Angelakis K. A.. (2010). Spermidine oxidase-derived H_2_O_2_ activates downstream Ca^2^+ channels which signal pollen tube growth. Plant J. 63, 1042–1053. 10.1111/j.1365-313X.2010.04301.x, PMID: 20626657

[ref141] Wu J. Shu S. Li C. Sun J. Guo S. (2018). Spermidine-mediated hydrogen peroxide signaling enhances the antioxidant capacity of salt-stressed cucumber roots. Plant Physiol. Biochem. 128, 152–162. 10.1016/j.plaphy.2018.05.002, PMID: 29778839

[ref142] Wuddineh W. Minocha R. Minocha S. C. (2018). Polyamines in the context of metabolic networks. Methods Mol. Biol. 1694, 1–23. 10.1007/978-1-4939-7398-9_1 29080151

[ref143] Yin J. Jia J. Lian Z. Hu Y. Guo J. Huo H.. (2019). Silicon enhances the salt tolerance of cucumber through increasing polyamine accumulation and decreasing oxidative damage. Ecotoxicol. Environ. Saf. 169, 8–17. 10.1016/j.ecoenv.2018.10.105, PMID: 30412897

[ref144] Yoda H. Fujimura K. Takahashi H. Munemura I. Uchimiya H. Sano H. (2009). Polyamines as a common source of hydrogen peroxide in host- and nonhost hypersensitive response during pathogen infection. Plant Mol. Biol. 70, 103–112. 10.1007/s11103-009-9459-0, PMID: 19190986

[ref145] Yoda H. Hiroi Y. Sano H. (2006). Polyamine oxidase is one of the key elements for oxidative burst to induce programmed cell death in tobacco cultured cells. Plant Physiol. 142, 193–206. 10.1104/pp.106.080515, PMID: 16844838 PMC1557616

[ref146] Yoda H. Yamaguchi Y. Sano H. (2003). Induction of hypersensitive cell death by hydrogen peroxide produced through polyamine degradation in tobacco plants. Plant Physiol. 132, 1973–1981. 10.1104/pp.103.024737, PMID: 12913153 PMC181282

[ref147] Yu Y. Zhou W. Zhou K. Liu W. Liang X. Chen Y.. (2018). Polyamines modulate aluminum-induced oxidative stress differently by inducing or reducing H_2_O_2_ production in wheat. Chemosphere 212, 645–653. 10.1016/j.chemosphere.2018.08.133, PMID: 30173111

[ref148] Zarei A. Trobacher C. P. Cooke A. R. Meyers A. J. Hall J. C. Shelp B. J. (2015). Apple fruit copper amine oxidase isoforms: peroxisomal MdAO1 prefers diamines as substrates, whereas extracellular MdAO2 exclusively utilizes monoamines. Plant Cell Physiol. 56, 137–147. 10.1093/pcp/pcu155, PMID: 25378687

[ref149] Zarepour M. Kaspari K. Stagge S. Rethmeier R. Mendel R. R. Bittner F. (2010). Xanthine dehydrogenase AtXDH1 from *Arabidopsis thaliana* is a potent producer of superoxide anions via its NADH oxidase activity. Plant Mol. Biol. 72, 301–310. 10.1007/s11103-009-9570-2, PMID: 19915948

[ref150] Zarza X. Atanasov K. E. Marco F. Arbona V. Carrasco P. Kopka J. (2017). Polyamine oxidase 5 loss-of-function mutations in *Arabidopsis thaliana* trigger metabolic and transcriptional reprogramming and promote salt stress tolerance. Plant Cell Environ. 40, 527–542. 10.1111/pce.12714 26791972

[ref151] Zepeda-Jazo I. Pottosin I. (2018). Methods related to polyamine control of cation transport across plant membranes. Methods Mol. Biol. 1694, 257–276. 10.1007/978-1-4939-7398-9_23 29080173

[ref152] Zhuo C. Liang L. Zhao Y. Guo Z. Lu S. (2018). A cold responsive ethylene responsive factor from Medicago falcata confers cold tolerance by up-regulation of polyamine turnover, antioxidant protection, and proline accumulation. Plant Cell Environ. 41, 2021–2032. 10.1111/pce.13114, PMID: 29216408

[ref153] Ziosi V. Bregoli A. M. Fregola F. Costa G. Torrigiani P. (2009). Jasmonate-induced ripening delay is associated with up-regulation of polyamine levels in peach fruit. J. Plant Physiol. 166, 938–946. 10.1016/j.jplph.2008.11.014, PMID: 19185952

